# Metal Removal from Nickel-Containing Effluents Using Mineral–Organic Hybrid Adsorbent

**DOI:** 10.3390/ma13194462

**Published:** 2020-10-08

**Authors:** Inga Zinicovscaia, Nikita Yushin, Dmitrii Grozdov, Konstantin Vergel, Nadezhda Popova, Grigoriy Artemiev, Alexey Safonov

**Affiliations:** 1 Joint Institute for Nuclear Research, Joliot-Curie Str. 6, 1419890 Dubna, Russia; ynik_62@mail.ru (N.Y.); grozdov@jinr.ru (D.G.); verkn@mail.ru (K.V.); 2 Horia Hulubei National Institute for R&D in Physics and Nuclear Engineering, 30 Reactorului, MG-6 Bucharest-Magurele, Romania; 3 Frumkin Institute of Physical Chemistry, Russian Academy of Science, 31 Leninsky Prospect, GSP-1, 119071 Moscow, Russia; no.hope996@gmail.com (N.P.); artemyev56@gmail.com (G.A.); alexeysafonof@gmail.com (A.S.)

**Keywords:** adsorption, hybrid adsorbent, industrial effluent, pollution, *Shewanella xiamenensis*, wastewater treatment

## Abstract

Nickel is one of the most dangerous environmental pollutants and its removal from wastewater is an important task. The capacity of a mineral–organic hybrid adsorbent, consisting of *Shewanella xiamenensis* biofilm and zeolite (clinoptilolite of the Chola deposit), to remove metal ions from nickel-containing batch systems under different experimental conditions was tested. The obtained biosorbent was characterized using neutron activation, SEM, and FTIR techniques. It was established that maximum removal of cations, up to 100%, was achieved at pH 6.0. Several mathematical models were applied to describe the equilibrium and kinetics data. The maximum adsorption capacity of the hybrid biosorbent, calculated using the Langmuir model, varied from 3.6 to 3.9 mg/g. Negative Gibbs energy values and positive *∆H*° values indicate the spontaneous and endothermic character of the biosorption process. The effects of several parameters (pH and biosorbent dosage) on Ni(II) removal from real effluent, containing nickel with a concentration of 125 mg/L, were investigated. The optimal pH for Ni(II) removal was 5.0–6.0 and an increase of sorbent dosage from 0.5 to 2.0 led to an increase in Ni(II) removal from 17% to 27%. At two times effluent dilution, maximum Ni(II) removal of 26% was attained at pH 6.0 and sorbent dosage of 1.0 g. A 12-fold effluent dilution resulted in the removal of 72% of Ni(II) at the same pH and sorbent dosage values. The obtained hybrid biosorbent can be used for Ni(II) removal from industrial effluents with low Ni(II) concentrations.

## 1. Introduction

Nickel compounds are one of the most dangerous environmental pollutants. The release of nickel-containing effluents into the environment can cause severe soil and water pollution [1]. Nickel is used in a wide range of applications in numerous industrial processes, the most important of which are mineral processing, electroplating, and production of paints, chemicals, and batteries [1,2].

Traditional techniques such as ion-exchange, membrane filtration, chemical precipitation, and adsorption are used for nickel removal from wastewater. The application of these techniques is limited due to the resulting generation of substantial volumes of sludge, which leads to secondary environmental pollution, as well as the high cost (including sludge disposal), high energy requirements, and impracticality at low metal concentrations in wastewater [2,3]. Biosorption using dry or alive microorganisms can be considered as an alternative to conventional techniques [2]. 

Among the microorganisms used as biosorbents, bacteria have an important role due to their ubiquity, large surfaces, resistance to metal ions at high concentrations, and the capability to reduce metal ions to less toxic forms. Often, biofilms alone show low adsorption capacity and their removal from treated effluents is a difficult task. To face this challenge, the immobilization of bacteria onto cheap, porous materials is a suitable option [4,5]. Zeolites are excellent ion exchangers which are characterized by a structural negative charge, which makes them perfect candidates for the removal of cations. Zeolites are readily available materials possessing excellent thermal and radiation stability and are characterized by a three-dimensional tetrahedral network and uniform porous structure [6]. At the same time, it should be mentioned that zeolites possess a low affinity for anions. The application of hybrid biosorbents (zeolite and bacteria) can improve the removal capacity for heavy metals, including anions [7].

An *Escherichia coli* biofilm placed on zeolite was applied for Cu(II) and Zn(II) ion removal from aqueous solutions [4]. Quintelas et al. [8] studied the efficiency of chromium(VI), cadmium, nickel, and iron removal by *Escherichia coli* biofilm supported on zeolite NaY. The biosorption behavior of a biofilm of *Arthrobacter viscosus* placed on 13 X zeolite towards Ni(II) was investigated by Lameiras et al. [9]. *Arthrobacter viscosus* supported on NaY zeolite was applied for dye and Cr(VI) removal [7]. The bacterial biofilm of *Arthrobacter viscosus* placed on activated carbon and natural zeolite was used to adsorb Cr (VI) from solutions [10]. The possibility of using a biofilm of *Shewanella xiamenensis* placed on zeolite for the treatment of complex chromium-containing effluents was reported by Zinicovscaia et al. [5].

In the present study, the biosorption capacity of a hybrid adsorbent, which consisted of *Shewanella xiamenensis* biofilm and zeolite, for the treatment of synthetic and real nickel-containing effluents under different experimental conditions was investigated. Adsorption equilibrium data were described using Langmuir, Freundlich, and Temkin models. The kinetics data were analyzed using pseudo-first, pseudo-second-order, Elovich, and Webber Morris models. The thermodynamic parameters of the biosorption were investigated. The effect of pH, sorbent dosage, and effluent dilution on Ni(II) removal from real effluent was assessed.

## 2. Materials and Methods

### 2.1. Chemicals

Chemicals Ni(NO_3_)_2_·6H_2_O, CuSO_4_, Zn(NO_3_)_2_·6H_2_O, Sr(NO_3_)_2_, Na_2_MoO_4_·2H_2_O, CrO_3_, HNO_3_, and NaOH used in this study were obtained from Sigma-Aldrich (Darmstadt, Germany) and were of analytical grade. Working solutions were prepared using demineralized water.

### 2.2. Synthetic Effluents

According to literature data, nickel concentrations in the main part of industrial effluents usually do not exceed 10 mg/L [11,12,13,14], with some exceptions [14,15]. In the present study, four synthetic effluents, Ni(II), Ni(II)-Sr(II)-Cu(II)-Zn(II), Ni(II)-Cr(VI)-Fe(III), Ni(II)-Zn(II)-Mo(VI)-Cu(II), were prepared (Table 1).

### 2.3. Industrial Effluent Characterization

The complex nickel-containing effluent was received from an electroplating company (Dubna, Russia) (Table 2).

### 2.4. Preparation of Biosorbent

As biosorbent bacteria, *Shewanella xiamenensis* DCB2-1 isolated from nitrate- and radionuclide-contaminated groundwater was used [16]. Shewanellae are members of the γ-proteobacteria facultative anaerobes, which were isolated from different environments [17]. Shewanella species possess good metal bioaccumulation and bioreduction capacities [18,19,20,21], while information about their biosorption capacity is very limited [5,22].

Zeolite (i.e., clinoptilolite) used in the present study was purchased from the “Zeolite-Trade” company (Moscow, Russia). Prior to the experiments, it was dried, granulated, and sieved. The zeolite chemical composition according to the manufacturer is Al_2_O_3_—12.9–13.2%; SiO_2_—66.2–78.3%; K_2_O—4.0–4.8%; Na_2_O—1.8-2.2%; CaO—1.8–2.4%; Fe_2_O_3_—0.8–1.2%; H_2_O—10–12%, V, Mn, Cu, Be, Rb, and As less than 0.03%. It represents isometric aggregates of 3-5 mm, consisting of microaggregates of micron size with thin isometric pores and elongate channels [5]. 

The fabrication process of the hybrid biosorbent proceeded in the following way: the bacteria *Shewanella xiamenensis* DCB2-1 were grown aerobically for three days in a culture medium containing mineral salts in the following concentrations (g/L): K_2_HPO_4_—1.5; KH_2_PO_4_—0.75; NH_4_Cl—0.3; NaCl—5.0; MgSO_4_·7H_2_O—0.1; KCl—0.1; CaCl_2_—0.02 at pH 7.0. On the third day of biomass growth, 50 g of zeolite, 300–100 µm fraction, was added to the cultivation medium, and the biomass was cultivated until the 7th day. During the experiment, flasks with biomass were stirred daily and the cultivation medium pH remained constant. After 7 days, the biomass was filtered, freeze-dried, and used for further biosorption experiments.

### 2.5. Metal Removal from Synthetic Effluents

To examine the sorption capacity of the hybrid adsorbent, 0.5 g of biosorbent was added to 50 mL of synthetic solution and continuously agitated for 120 min at 200 rpm. The pH of the experimental solutions varied from 2.0 to 6.0. To obtain necessary pH values, NaOH or HNO_3_ were used. For kinetic studies, samples were withdrawn at certain periods of time 15, 30, 60, 90, 120, 150, and 180 min. For equilibrium studies, the nickel concentration in the solution ranged from 10 to 100 mg/L, while concentrations of other metal ions were kept at the same level. The thermodynamic studies were performed in the temperature range from 20 to 50 °C. All experiments were performed in triplicate and the mean values were used in further discussion.

### 2.6. Metal Removal from Real Effluent

In the experiments with real effluent, the effect of pH (2.0–6.0), sorbent dosage, and effluent dilution on Ni(II) removal was investigated. To study the effect of the adsorbent dosage on the removal efficiency, 0.5–2.0 g of hybrid adsorbent was added to 100 mL of effluent at pH 6.0. In the next experiment, the initial effluent was diluted 2 and 12 times and then 0.5 or 1.0 g of hybrid adsorbent was added. In all experiments, the suspensions were shaken at 200 rpm for 120 min, then the sorbent was removed by filtration using a 0.45 µm syringe filter from Sigma-Aldrich (Darmstadt, Germany).

The metal uptake *q* (mg/g) was counted according to Formula (1):

(1) $$q=\frac{V\left(C_{i}-C_{f}\right)}{m}$$

and removal efficiency, *E* (%), according to Equation (2):

(2) $$E=\frac{C_{i}-C_{f}}{C_{i}}\times 100$$

where *V* is the volume of the solution, mL; *C_i_* and C_f_ are the initial and final metal concentrations, mg/L, and *m* is the mass of sorbent, g.

### 2.7. Methods

To determine the elemental content of natural zeolite and hybrid sorbent and to assess the effectiveness of metal sorption from batch solutions, samples of hybrid adsorbent were subjected to neutron activation analysis (NAA) under a pulsed fast reactor IBR-2 (JINR, Dubna, Russia). Before analysis, biosorbent samples were dried and packed in aluminum cups. Afterward, they were irradiated for 3 days at the neutron flux of 1.8 × 10^11^ cm^−2^ s^−1^, repacked, and measured after 4 and 20 days. The samples were irradiated simultaneously with standard reference materials. The analysis of the spectra was performed using the Genie2000 software by Canberra, and element concentrations were determined using the software “Concentration”. The difference between experimental values and certified values was in the range of 2–10%. Copper concentration in the experimental solutions was determined following the procedure described in Zinicovscaia et al. [5]. To determine metal concentrations in the real effluent, the Element 2™ High-Resolution ICP-MS systems (The Thermo Scientific, Chemnitz, Germany) were used. The characteristics of the device are given in Kuznetsova et al. [23]. Metal concentrations in effluent were measured immediately after the sorption experiments.

Microbial biofilm formation on the zeolite surface was confirmed using the confocal laser scanning microscope Leica SP5 (Leica, Berlin, Germany) and characterization of the porous texture of the biosorbent was carried out using AUTOSORB 1MP (Quantachrome Instruments, Amsterdam, The Netherlands). The images were examined using the Imaris 7.0.0 (Bitplane, Zurich, Switzerland) to calculate the percentage of area covered by bacterial cells and polysaccharides. An argon laser with wavelengths of 488 and 594 nm was applied for detecting wheat germ agglutinin (WGA) fluorescence and cell-permeant SYTO 11 green fluorescent nucleic acid stain, respectively. Details of the analysis are presented in Zinicovscaia et al. [5].

Fourier-transform infrared spectroscopy (FTIR) was used to reveal the role of surface functional groups in binding metal ions. Spectra were recorded using the Nicolet 6700 spectrometer (Thermo Scientific, Waltham, MA, USA). Zeta potential of the hybrid adsorbent at the pH range of 2.0–6.0 was measured using Zetasizer Nano ZSP (Malvern Instruments, Malvern, UK).

## 3. Results

### 3.1. Biosorbent Characterization

**Figure 1:**
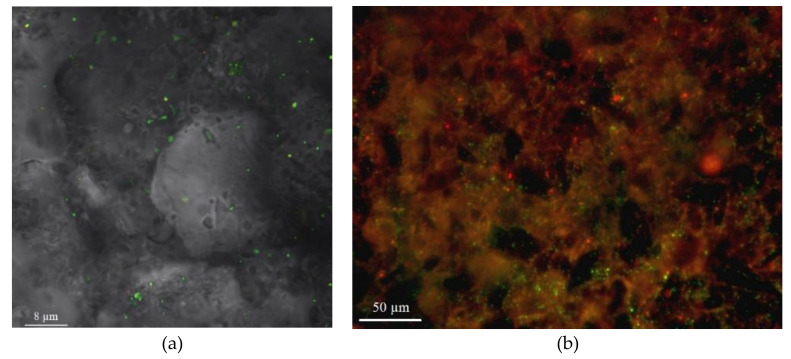
Zeolite surface before (**a**) and after (**b**) biofilm formation.

According to the results of the confocal laser scanning microscope, 81.6% of the surface of zeolite was covered by biofilm and 18.4% of the surface remained uncoated. The biofilm consisted of 74.3% polysaccharides and 7.3% bacterial cells (Figure 1).

**Figure 2:**
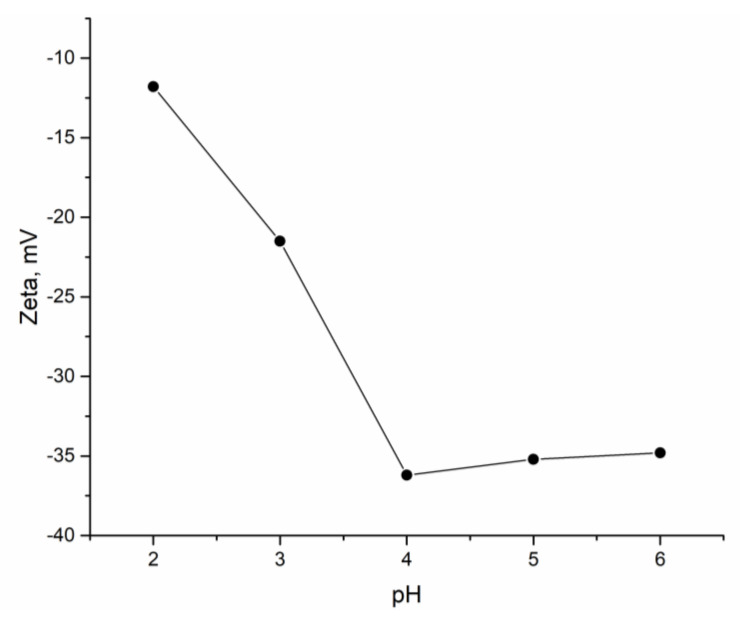
Zeta potential of hybrid biosorbent as a function of pH.

The zeta potential of the hybrid biosorbent was measured in the pH range 2.0–6.0 to determine the charge of the biosorbent. According to the obtained results (Figure 2), the zeta potential of the hybrid biosorbent was negative at the analyzed range of pH. The specific surface areas of the hybrid biosorbent determined in our previous study showed it to be 34 m²/g, with dominant pore sizes of 3.0, 5.5, and 7.5 nm [5].

Neutron activation analysis was applied to determine the chemical composition of the natural zeolite and hybrid adsorbent. Data presented in Table 3 show that modification of the zeolite surface leads to a change in the elemental composition. From 30 elements determined in the natural zeolite, only 16 elements were detected in the hybrid adsorbent. After biofilm formation on the zeolite surface, it lost a significant amount of Al and Si, which indicated structural changes in its surface layer. Elements such as Sr, Rb, Sb, Ba, Cs, Ce, Eu, Gd, Tb, Yb, Hf, and Th were determined only in the zeolite, which may be explained by their release in the nutrient medium. The release of Na was associated with uptake of K, Ca, and Mg. The increase in the content of some elements in the hybrid adsorbent can be explained by their sorption from the cultivation medium.

**Figure 3:**
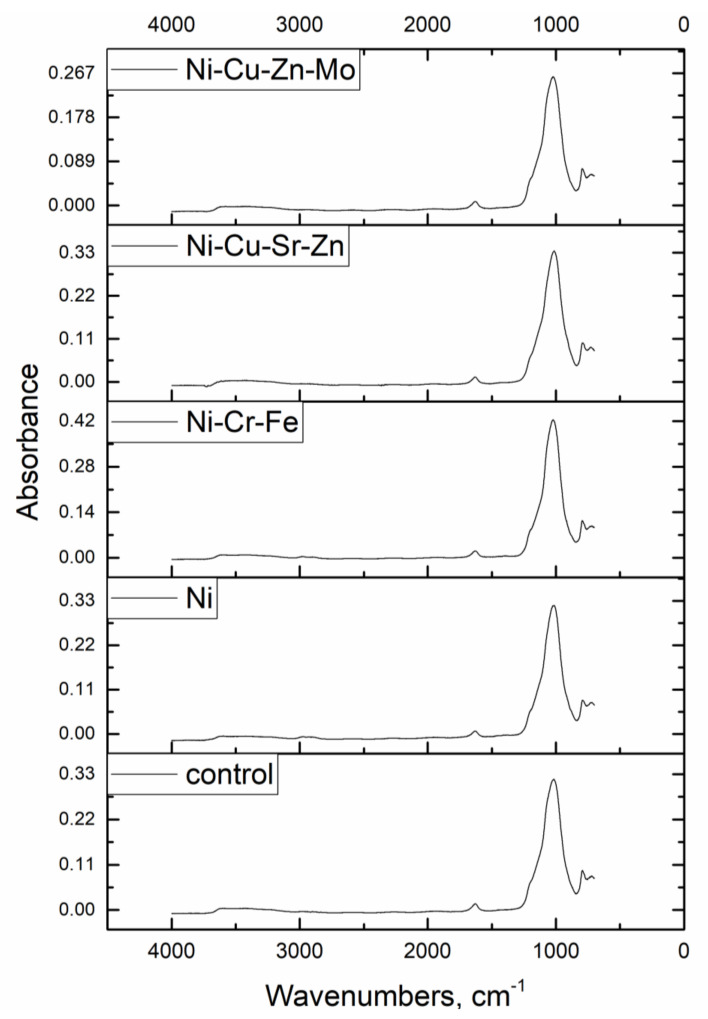
FTIR spectra of control and metal-loaded hybrid adsorbent.

The surface functional groups of the hybrid adsorbent and metal-loaded adsorbent determined by FTIR spectroscopy are illustrated in Figure 3. Broad bands at wavenumber region 3650–3200 cm^−1^ and deformation at area 1650–1600 cm^−1^ could be attributed to −OH stretching [24]. It could also be explained by the hydrophilic nature of zeolite that can adsorb water molecules through hydrogen bonding [25], as well as to hydroxyl functional groups of the biofilm layer formed on the zeolite surface. A wide adsorption belt at wavenumbers from 3000 to 2850 cm^−1^ is due to C–H stretching vibrations of the bacterial biofilm. The intensive deformation bands in the range of 1200–750 cm^−1^ are assigned to internal Si–O(Si) and Si–O(Al) vibrations in tetrahedra or aluminum and silico-oxygen bridges. The 3D covalent arrangement of non-tetrahedral cations into alumino-silicate frameworks was observed below 700 cm^−1^ due to pseudo-lattice vibrations of structural units [24,26]. The band positions in the IR spectra of the metal-loaded adsorbent were slightly shifted but a significant change in the band intensities of −OH, C–H, Si–O(Si), and Si–O(Al) groups after metal ion sorption was not observed.

### 3.2. Metal Removal from Synthetic Effluents

#### 3.2.1. Effect of pH on Metal Ion Removal

**Figure 4:**
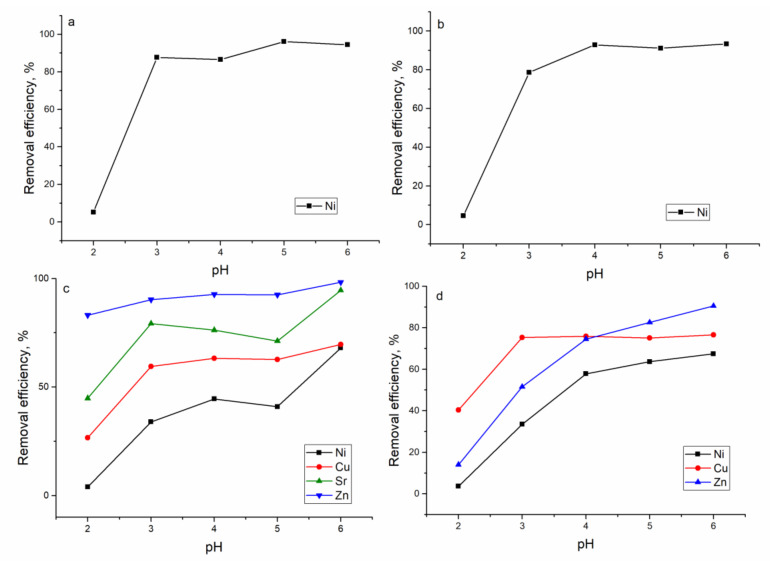
Effect of pH on metal removal by hybrid adsorbent: (**a**) Ni(II), (**b**) Ni(II)-Cr(VI)-Fe(III), (**c**) Ni(II)-Sr(II)-Cu(II)-Zn(II), and (**d**) Ni(II)-Zn(II)-Mo(VI)-Cu(II).

The thermodynamic speciation of metal ions in analyzed systems as a function of pH is presented in Figures S1–S4. The database of thermodynamic data llnl.dat of calculation code PhreeqC 2.18 was used for simulation. Metal removal from the analyzed systems showed it to be pH-dependent and *Shewanella xiamenensis* biofilm placed on zeolite showed it to be an efficient biosorbent only in the case of elements present in the solution in cationic form. The removal of Ni(II) in all analyzed systems increased with the increase in pH, and at pH 6.0, the following values were achieved: 94% in Ni(II) and Ni(II)-Cr(VI)-Fe(III) systems; 68% in Ni(II)-Sr(II)-Cu(II)-Zn(II) and Ni(II)-Zn(II)-Mo(VI)-Cu(II) systems (Figure 4).

The biosorbent also showed high removal capacity for Cu(II), Zn(II), and Sr(II). Maximum Sr(II) removal was attained at pH 6.0 and constituted 95 %. The optimal pH for Cu(II) removal in the Ni(II)-Sr(II)-Cu(II)-Zn(II) system was 6.0 when 70% of Cu(II) was sorbed from the solution, and in Ni(II)-Zn(II)-Mo(VI)-Cu(II), pH in the range 3.0–6.0 was favorable for Cu(II) removal. More than 90% of Zn(II) was removed from the systems containing this element. Removal of metal ions present in solutions in an anionic form (Cr(VI), Mo(VI)) and Fe(III)) was not achieved. Since Ni(II) was the element of main interest, further experiments were performed at pH 6.0.

#### 3.2.2. Kinetic Studies

**Figure 5:**
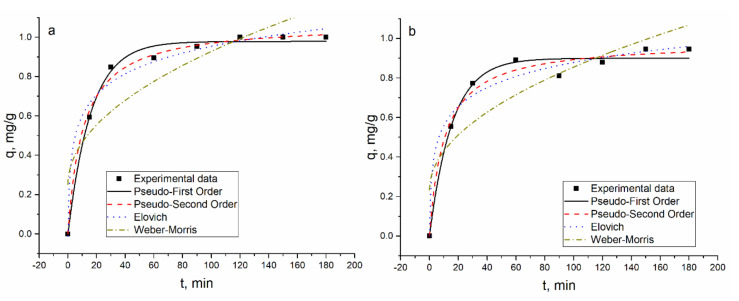
Ni(II) adsorption on hybrid adsorbent in the (**a**) Ni(II) and (**b**) Ni(II)-Cr(VI)-Fe(III) systems.

The biosorption of metal ions by *Shewanella xiamenensis* biofilm placed on zeolite was a quick process. In the Ni(II) system, in two hours, Ni(II) was completely removed from the solution (Figure 3) and in the Ni(II)-Cr(VI)-Fe(III) system, its maximum removal, 95%, was attained in 150 min (Figure 2). Subsequently, in both systems, equilibrium was reached (Figure 5). At pH 6.0, removal of Cr(VI) and Fe(III) did not occur.

**Figure 6:**
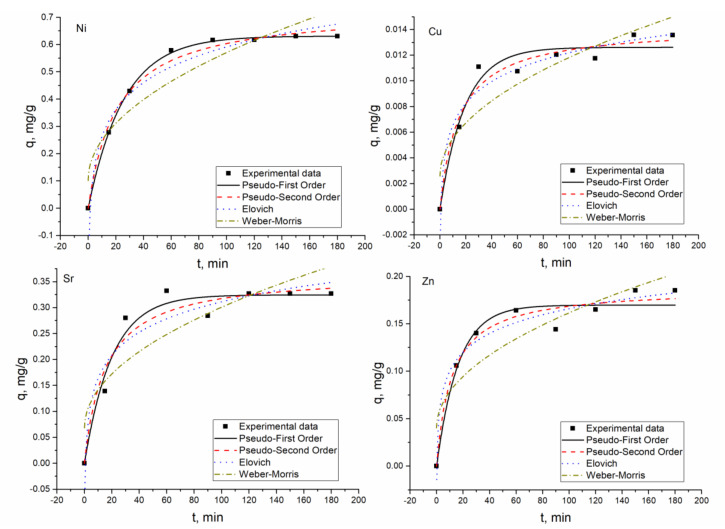
Metal adsorption on hybrid adsorbent in Ni(II)-Sr(II)-Cu(II)-Zn(II) system.

In the Ni(II)-Sr(II)-Cu(II)-Zn(II) and Ni(II)-Zn(II)-Mo(VI)-Cu(II) systems, 62% of Ni(II) was removed in 90 min and then its removal stabilized at the same level. In the Ni(II)-Sr(II)-Cu(II)-Zn(II) system, maximum Zn(II) and Cu(II) removal (98.5% and 74%, respectively) was achieved in 150 min, and maximum Sr(II) removal in 120 min (65%) (Figure 6).

**Figure 7:**
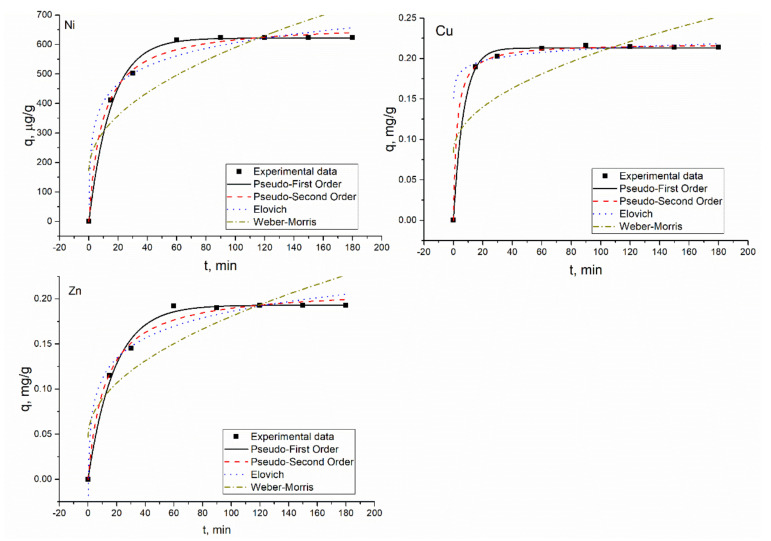
Metal adsorption on hybrid adsorbent in the Ni(II)-Zn(II)-Mo(VI)-Cu(II) system.

In the Ni(II)-Zn(II)-Mo(VI)-Cu(II) system (Figure 4), 60 min was enough for equilibrium to be attained for Cu(II) and Zn(II). The maximum removal of Cu(II) constituted 79% and of Zn(II) 96.5% (Figure 7). Mo(VI), which was present in the solution in anionic form, was not adsorbed onto the biosorbent.

Experimentally obtained data were described using four models, which are presented below, as Equations (3)–(6).

The pseudo-first-order model (PFO) is expressed by the following equation:

(3) $$q_{t}=\;q_{e\;}(1-e^{-k_{1}t)}$$

where *q_e_* and *q_t_* are the quantities of metal (mg/g) sorbed from the solution at equilibrium and at *t* (min) time, respectively, and *k*_1_ (1/min) is the pseudo-first-order rate constant. 

The pseudo-second-order model (PSO) is expressed by Equation (4):

(4) $$q=\frac{q_{e}^{2}k_{2}t}{1+q_{e}k_{2}t}$$

where *k*_2_ (g/mg·min) is the second-order rate constant.

The Elovich model (EM) is expressed by Equation (5):

(5) $$q_{t\;}=\;\frac{1}{\beta }ln\left(1+\alpha \beta t\right)$$

where *α* (g/mg∙min) and *β* (g/mg) are the equation constants.

The Weber and Morris intraparticle diffusion model (IPM) is expressed by Equation (6):

(6) $$q=\;k_{diff}\cdot t^{0.5}+C_{i}$$

where *k_diff_* is a rate parameter (mg/g·min^1/2^), and *C_i_* is the intercept, which relates to the thickness of the boundary layer.

The applicability of kinetic models was confirmed by *SSE* (sum of error squares), %:

(7) $$SSE=\frac{\sqrt{\sum \;{\left(q_{e,\;cal}-q_{e,\;exp}\right)}^{2}}}{N}$$

where *N* is the number of experimental points.

The experimentally obtained results and their theoretical description are presented in Figure 4, Figure 5, Figure 6 and Figure 7 and calculated coefficients are listed in Table 4. The q values, both calculated and experimental ones, for the PFO and PSO models were in good agreement. In Ni(II) and Ni(II)-Cr(VI)-Fe(III) systems, high coefficients of determination obtained for PFO and PSO models indicated their applicability for the description of experimental data. The *R*^2^ values showed that the adsorption of cations present in the Ni(II)-Sr(II)-Cu(II)-Zn(II) system obeyed different models: PSO for Ni(II) and Cu(II); EM for Zn(II); and PFO model for Sr(II). In the Ni(II)-Zn(II)-Mo(VI)-Cu(II) system, PFO and PSO models were found to be suitable for describing the experimentally obtained data for Ni(II), Zn(II), and Cu(II). The R^2^ values for EM in all systems were greater than 0.9, while those for IPM were significantly lower.

One more parameter calculated to prove the applicability of the models was adjusted *R* squared:

(8) $$R_{\mathrm{adj}}^{2}=1-\left(1-R^{2}\right)\left[\frac{n-1}{n-\left(k+1\right)}\right]$$

where *R*^2^ was obtained from the applied models, *K* is the number of predictors and *n* is the number of experimental values.

The similar values obtained for *R*^2^ and *R*^2^_adj_ indicated the applicability of the applied models in describing the experimentally obtained results.

#### 3.2.3. Equilibrium Study

The increase in Ni(II) concentration in the analyzed solutions up to 100 mg/L led to the increase in the biosorbent adsorption capacity from 0.9 to 2.9 mg/g in the Ni(II) system, from 0.8 to 2.8 mg/g in the Ni(II)-Cr(VI)-Fe(III) system, from 0.9 to 2.8 mg/g in the Ni(II)-Sr(II)-Cu(II)-Zn(II) system, and from 0.6 to 1.7 mg/g in the Ni(II)-Zn(II)-Mo(VI)-Cu(II) system. In the Ni(II)-Cr(VI)-Fe(III) system, adsorption of Cr(VI) and Fe(III) did not occur. In the Ni(II)-Sr(II)-Cu(II)-Zn(II) system, Zn(II) removal was not affected by the increase in Ni(II) concentration in the solution, while removal of Cu(II) and Sr(II) increased by 12% and 10%, respectively. In the Ni(II)-Zn(II)-Mo(VI)-Cu(II) system, Cu(II) removal increased by 10 % with the increase in Ni(II) concentration from 10 to 100 mg/L, while Zn(II) removal continuously decreased.

Three models, Langmuir (9), Freundlich (10), and Temkin (11), were applied for equilibrium modeling.

(9) $$q_{e}=\frac{q_{m\;}bC_{e}}{1+bC_{e}}$$

(10) $$q_{e}=K_{F}Ce^{\frac{1}{n}}$$

(11) $$q_{e}=\frac{RT}{b_{T}}ln\left(a_{T}C_{e}\right)$$

where *C_e_* is equilibrium concentration (mg/L), *q_m_* is maximum adsorption capacity (mg/g), *b* is Langmuir adsorption constant (L/mg), *K_F_* and *n* are Freundlich equation constants, 1/*b_T_* manifests the sorption potential of the sorbent, *a_T_* is Temkin constant, *R* is the universal gas constant (8.314 J K^−1^ mol^−1^) and *T* is the temperature (K) [27].

Separation factors *R_L_* calculated according to Formula (12) explained the favorability of the adsorption process.

(12) $$R_{L}=\frac{1}{1+bC_{0}}$$

**Figure 8:**
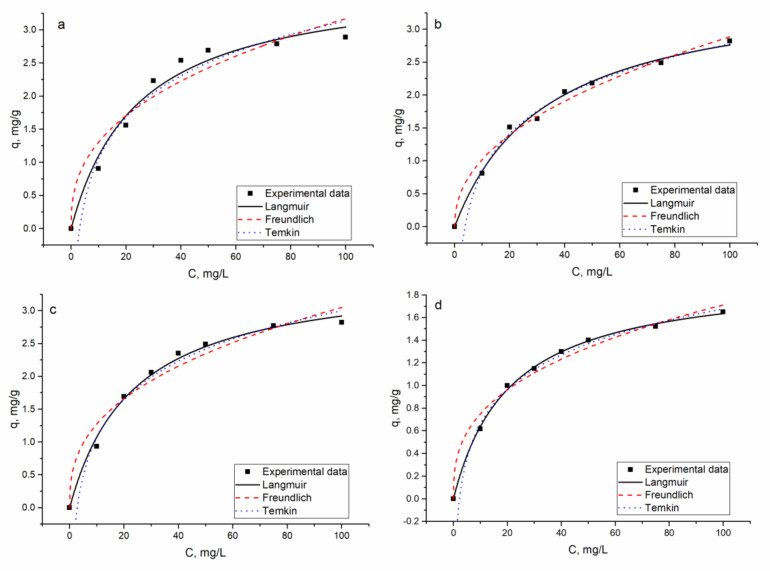
The adsorption isotherms for Ni(II) ion removal on hybrid adsorbent: (**a**) Ni(II), (**b**) Ni(II)-Cr(VI)-Fe(III), (**c**) Ni(II)-Sr(II)-Cu(II)-Zn(II), and (**d**) Ni(II)-Zn(II)-Mo(VI)-Cu(II).

The Langmuir, Freundlich, and Temkin isotherm constants were calculated by nonlinear regression and are presented in Figure 8 with the experimental data. The obtained parameters are listed in Table 5.

According to *R*^2^ values, the Langmuir and Temkin isotherms were suitable to describe the experimentally obtained data. The coefficient of determination obtained for the Freundlich model was smaller; however, the Freundlich constant 1/*n* was less than 1.0 in all analyzed systems, confirming favorable adsorption. The maximum adsorption capacity of the biosorbent in Ni(II), Ni(II)-Sr(II)-Cu(II)-Zn(II), and Ni(II)-Cr(III)-Fe(III) systems had very similar values, while in the Ni(II)-Zn(II)-Mo(VI)-Cu(II) system, it was almost two times smaller. This is in agreement with experimentally obtained data. The separation factors *R_L_* values lower than 1.0 indicate favorable Ni(II) biosorption.

#### 3.2.4. Thermodynamic Studies

**Figure 9:**
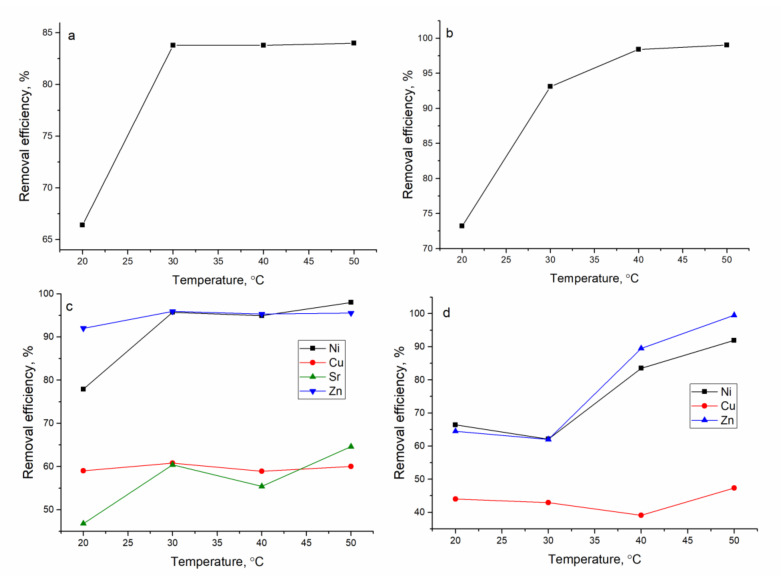
Effect of temperature on metal removal by hybrid adsorbent: (**a**) Ni(II), (**b**) Ni(II)-Cr(VI)-Fe(III), (**c**) Ni(II)-Sr(II)-Cu(II)-Zn(II), and (**d**) Ni(II)-Zn(II)-Mo(VI)-Cu(II).

An increase in the temperature in all analyzed systems had a positive effect on Ni(II) removal efficiency (Figure 9). In Ni(II)-Sr(II)-Cu(II)-Zn(II) and Ni(II)-Cr(III)-Fe(III) systems, a temperature increase up to 50 °C resulted in the removal of 99% of Ni(II) from the solution. In the other two systems, the removal was also very high, at the level of 90%. Copper removal in Ni(II)-Sr(II)-Cu(II)-Zn(II) and Ni(II)-Zn(II)-Mo(VI)-Cu(II) systems was not affected by the temperature increase. In the Ni(II)-Sr(II)-Cu(II)-Zn(II) system, the removal of Zn(II) was irrespective of the temperature increase, while Sr(II) increased by 20% with a temperature increase from 20 to 50 °C. Zn(II) removal in the Ni(II)-Zn(II)-Mo(VI)-Cu(II) system increased by 35% at a temperature of 50 °C in comparison to removal at 20 °C.

The thermodynamic parameters ΔG°, ΔH°, and ΔS° were calculated according to Equations (13)–(15):

(13) $$\ln K_{d}=\frac{\Delta S^{0}}{R}-\frac{\Delta H^{0}}{RT}$$

(14) $$\Delta G^{0}\;=\;\Delta H^{0}\;-\;T\Delta S^{0}$$

where *K_d_* is the distribution coefficient and it is calculated according to Equation (15):

(15) $$K_{d}=\frac{\left(C_{0}-C_{e}\right)V}{mC_{e}}$$

From the plot of ln*K_d_* versus 1/*T* (Figure S5), enthalpy and entropy values were calculated and their values are given in Table 6.

The Gibbs free energy was negative for all metal ions present in analyzed systems in the cationic form, indicating the spontaneous character of the biosorption process. The enthalpy and entropy values were positive for cations. Positive values of ΔH° showed that metal biosorption was an endothermic process.

### 3.3. Ni(II) Removal from Industrial Effluent

**Figure 10:**
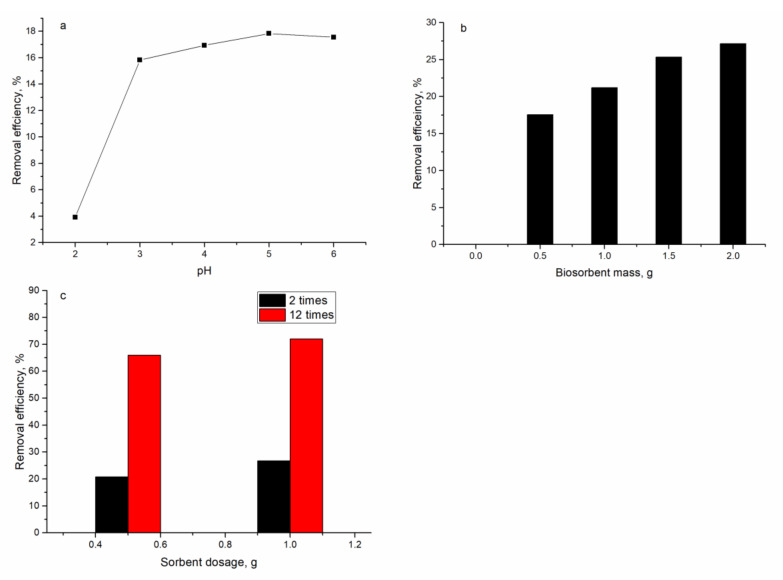
Effect of different parameters on Ni(II) removal from industrial effluent: (**a**) pH, (**b**) sorbent dosage (initial effluent), (**c**) sorbent dosage (diluted effluent).

The effluent taken from a galvanic unit contained Ni(II) in a concentration of 125 mg/L, as well as Zn, Cr, Fe, Mo, Cu, and Sr in significantly lower concentrations (below maximum permissible limits). In the experiments with industrial effluent, firstly, the effect of pH on the Ni(II) removal was investigated. The pH of the effluent varied from 2.0 to 6.0 and the sorbent dosage was 0.5 g. In comparison with the batch experiments, removal of Ni(II) from real effluent was significantly lower (Figure 10a). At pH 2, it constituted only 3.0% and with an increase in the pH up to 6.0, it rose to 17%. To improve Ni(II) removal from effluent, in the next experiment, pH was maintained at 6.0 and the amount of biomass varied from 0.5 to 2.0 g (Figure 10b). The increase in the biosorbent mass resulted in increased Ni(II) removal, from 17% (sorbent dosage 0.5 g) to 27% (at sorbent dosage 2.0 g). Thus, the four-fold increase in sorbent mass caused Ni(II) removal to improve only by 10%. Since the concentration of Ni(II) in the effluent was very high, in the next experiment, the effluent was diluted 2 and 12 times with distilled water, and the effect of biosorbent dosage (0.5 and 1.0 g) on Ni(II) removal was assessed. According to the obtained data (Figure 10c), at sorbent dosage of 1.0 g, 26% of Ni(II) was removed from the solution; however, Ni(II) removal after 12-fold dilution amounted to 66% at a sorbent dosage of 0.5 g and to 72% at a dosage of 1.0 g.

## 4. Discussion

A study on the effect of pH on the sorption capacity of sorbent showed that the obtained hybrid adsorbent (*Shewanella xiamenensis* biofilm placed on zeolite) was effective for metal cation removal. The highest removal of Ni(II) was achieved in Ni(II) and Ni(II)-Cr(VI)-Fe(III) systems, in which there was no competition of other metal cations with Ni(II) for binding sites. The negative zeta potential values obtained at the studied pH range created unfavorable conditions for the sorption of anions. According to the thermodynamic calculations (Figure S1), at the pH range 2–8.2, nickel was present in the solution in dissolved form, while at pH > 8.2, low-soluble Ni(OH)_2_ was formed. In the case of the studied hybrid adsorbent, it is suggested that Ni(II) can interact with functional groups of *Shewanella xiamenensis*, as well as with zeolite. In our previous research, it was shown that at Ni(II) concentration in a solution of 10–14 mg/L, removal by cyanobacteria did not exceed 66% [28]. Bacteria showed higher Ni(II) removal and up to 80% of Ni(II) was removed by the species *Pseudomonas cepacian* 120S and *Bacillus subtilis* 117S from a solution at pH 7.0 [29].

Hydroxyl, carboxyl, carbonyl, and amino groups play an important role in Ni(II) binding by microorganisms [2,15]. Zeolites are minerals in which the process of chemical immobilization of heavy metals is based on the exchange of the alkali and alkaline earth metal cation with heavy metals [5]. FTIR analysis showed that biofilm functional groups were not involved in metal ion trapping. Thus, ion exchange can be proposed as the main mechanism of metal ion uptake from the analyzed solutions. Data obtained for Na and K using neutron activation analysis confirmed this fact since their content in the metal-loaded adsorbent decreased by 2–18% in respect to the control adsorbent. The theoretical exchange capacity of zeolite for Ni(II) was 4–11 times higher than the actual exchange capacity [30].

In a study by Handley-Sidhu et al [31], it was shown that the biofilm was responsible for less than 30% of Sr^2+^ sorption by biogenic hydroxyapatite. Ni(II) removal by *Arthrobacter viscosus* supported on zeolite 13 X constituted 94.1%. Authors showed that Ni(II) removal by zeolite was only faster than that by bacteria, suggesting that the main role in the removal of nickel belongs to zeolite, while bacteria slightly improve it [9]. Experiments performed on zeolite and on *Shewanella xiamenensis* separately (data not shown) demonstrated that zeolite was able to remove 70% of Ni(II) ions from the Ni(II) system; however, equilibrium was attained in 24 h. *Shewanella xiamenensis* removed 60% of Ni(II) in 30 min and then equilibrium was attained. Thus, it can be concluded that the main role in metal removal from the analyzed systems belonged to zeolite and bacterial biofilm accelerated the metal sorption process.

In the Ni(II)-Cr(III)-Fe(III) system, at the studied pH range, nickel was present in the solution in dissolved form and the chromium dominant form was hydrochromate ions, HCrO_4_^−^ (Figure S2). Iron(III) started to form a solid mineral phase at pH > 4.2. Ni(II) was almost completely removed in this system, while Cr(VI) and Fe(II) removal did not occur. According to literature data, it is known that Cr(VI) and Fe(II) are efficiently removed from solution at low pH values [27,32]. Low removal of Cr(VI) and Fe(II) by the studied biosorbent can be explained by possible biofilm disruption at acidic pH and the negative charge of the hybrid adsorbent, as zeta potential data showed.

In the other two systems, Ni(II) removal decreased almost by 30% in comparison with the one previously described. This can be associated with the presence of other metal cations in the analyzed systems and their competition for binding sites. In the Ni(II)-Cu(II)-Sr(II)-Zn(II) system, metal ions are present in the solution in dissolved form at the studied pH range (Figure S3). The preference of biosorbent for the analyzed elements changed in the following order: Zn > Sr > Cu > Ni. The adsorption capacity of zeolite for lead was significantly higher than for nickel [30]. Potassium content in the metal-loaded sorbent decreased by 15% in comparison with the control, while Na content was not affected by the sorption process.

In the Ni(II)-Zn(II)-Mo(VI)-Cu(II) system, Zn(II) and Ni(II) were present in soluble forms. Molybdenum in the analyzed complex system is present in the solution in dissolved form at pH 2.0–3.0, and at the pH range of 3.0–6.0, it is present in the form of copper molybdate. Copper is present both in dissolved form and in precipitated form as molybdate, CuMoO_4_ (Figure S4). The presence of Mo(VI) in the analyzed system did not affect the removal of other metal cations, while its removal from the solution did not occur. Besides ion exchange, metal precipitation can be expected as another mechanism of metal removal.

As was previously mentioned, the removal of anions by zeolite is hampered by the negative charge of the material. The maximum removal of chromium by a biofilm of *Escherichia coli* supported on NaY zeolite was achieved at pH in the range 4.6–5.1, of iron at pH 2.7–3.5, and of nickel at pH 5.7–6.2 [8]. The maximum removal of copper (54.98 %) and zinc (57.32%) by *Escherichia coli* biofilm placed on zeolite was attained at pH values of 4.8–5.7 and 4.5–5.5, respectively [4]. In experiments described by refs. [5,7], it was possible to achieve 50% and 35% removal of Cr(VI) by bacterial biofilm supported by zeolite. In the presented studies, living bacteria were used, which resulted in Cr(VI) reduction to Cr(III) and further sorption. In the present study, dried biosorbent was used and only sorption processes took place. Molybdenum was adsorbed at pH 3.0 on zeolite as a FeOMoO_2_(OH)·2H_2_O inner-sphere complex [33].

The optimal time for maximum removal of metal ions from the analyzed systems varied from 60 to 150 min. Removal of Cr(VI) by natural zeolite was lower and lasted longer than removal by grape and olive wastes used as adsorbents [34]. Optimum nickel uptake by natural zeolite was achieved at a contact time of 56–68 min and a pH of 4.8–6 [1]. The equilibrium time for adsorption of copper and zinc on *Escherichia coli* biofilm placed on zeolite was achieved after 5 and 4 days, respectively [4].

According to the coefficients of determination of the applied models, the PFO, PSO, and EM were found to be applicable to describe experimentally obtained data. The applicability of PSO and EM showed that chemical absorption and ion exchange are the main mechanisms of metal sorption by the hybrid adsorbent [27]. The R^2^ values for most of the elements in the analyzed systems were similar and ESS were calculated additionally. The EES values for both models, PFO and PSO, were very similar, indicating that the used models fit the data well. Since the R^2^ and ESS values for both models were very close, adsorption rate values were used as criteria to determine the best fitting model. For all elements except Ni(II) in Ni(II)-Zn(II)-Mo(VI)-Cu(II), the adsorption rate values calculated for the PSO model were higher than for the PFO model, pointing to a higher rate of adsorption. The PSO model fits better Ni(II) biosorption on *Arthrobacter viscosus* biofilm supported on 13 X zeolite [9].

The increase in Ni(II) concentration in the analyzed solutions up to 100 mg/L led to increased biosorbent adsorption capacity, up to 2.9 mg/g in Ni(II), Ni(II)-Cr(VI)-Fe(III), and Ni(II)-Sr(II)-Cu(II)-Zn(II) systems, while in the Ni(II)-Zn(II)-Mo(VI)-Cu(II) system, it constituted only 1.7 mg/g. At the same time, the Ni(II) removal efficiency decreased as a result of the increase in its concentration in the solution. At low metal concentrations, there are enough binding sites on the sorbent surface, and at higher metal concentrations, the decrease in the absorption is explained by the saturation of the adsorption sites [1]. In Ni(II)-Cr(VI)-Fe(III) and Ni(II)-Sr(II)-Cu(II)-Zn(II) systems, Ni(II) adsorption was not affected by the presence of other metal ions in the solution. Since equilibrium experiments were performed at pH 6.0, optimal for Ni(II) removal, the removal of Cr(VI) and Fe(III) did not occur and the same was observed for Mo(VI) in the Ni(II)-Zn(II)-Mo(VI)-Cu(II) system. In the Ni(II)-Sr(II)-Cu(II)-Zn(II) system, Ni(II) sorption was similar to that of the Ni(II) system, indicating that metal ions present in the complex system did not affect its removal. The decrease in Na and K content in the metal-loaded adsorbent did not exceed 10%. The lowest adsorption of Ni(II) was in the Ni(II)-Zn(II)-Mo(VI)-Cu(II) system. It is important to mention that in this system, a significant reduction of Na (by 20%) and K (by 30%) took place, which may indicate their involvement in ion exchange. Cu(II) removal was almost unaffected by the increase in nickel concentration in the solution, while Zn(II) removal drastically decreased. At a nickel concentration in the solution of 10 mg/L, 99% of Zn(II) was removed from the solution but a 10-fold increase in nickel concentration led to a decrease in its removal up to 39%.

The Langmuir model, which assumes a monolayer sorption, fit well the data obtained for Ni(II). The maximum sorption capacity in three of four systems was on the level of 3.6–3.9 mg/g, while in the Ni(II)-Zn(II)-Mo(VI)-Cu(II) system, it was significantly lower (1.98 mg/g). The R^2^ values for the Freundlich model were lower. For the Temkin isotherm, R^2^ values were on the level of the Langmuir model and the constant (B) values related to the heat of adsorption were in the range 0.4–0.9 kJ/mol. Adsorption is considered a physical process if bonding energy is in the range of 5–40 kJ/mol and the chemical one in the energy range 40–800 kJ/mol. The low values of energy obtained in the Temkin model in the present study suggested weak ionic interaction between the sorbate and the sorbent [35].

The sorption capacity of the analyzed hybrid sorbent toward Ni(II) was compared with data available for other sorbents (Table 7). The maximum sorption capacity obtained in the present study was lower than the data presented in the main part of the research, except for El-Sadaawy et al.’s [36] study.

The rise in temperature in the experimental solutions resulted in growth of Ni(II), Sr(II), and Zn(II) removal, while Cu(II) removal was not dependent on the temperature. The temperature increase to 50 °C in all analyzed systems had a positive effect on Ni(II) removal efficiency. According to the Gibbs energy values, the biosorption in the analyzed systems was spontaneous physical sorption [40]. Positive *∆H*º values were obtained for all metal ions and indicated by the endothermic character of the biosorption. Positive *ΔS*° values acquired for cations in the analyzed systems suggested high affinity and the presence of a low energy barrier of metal adsorption processes using *Shewanella xiamenensis* biofilm placed on zeolite [41].

The dosage of biosorbent and pH are the key parameters for efficient metal ion removal from industrial effluents. In the studied effluent, Ni(II) concentration was 125 mg/L, concentrations of other metal ions were considerably lower, and they were excluded from further discussion. The maximum removal of Ni(II) was achieved at pH range 4.0–6.0 and amounted to 17%, which was considerably lower than values obtained in batch experiments. Ni(II) removal by natural zeolite from wastewater constituted 60%; however, the initial Ni(II) concentration was almost 35 times lower than in the present study [42]. One of the ways to increase metal ion removal is to increase the dosage of the adsorbent. The increase in sorbent dosage from 0.5 to 2.0 g resulted in a rise in Ni(II) removal only by 10%. The percent of Ni(II) adsorbed by coir pith from an effluent containing 145 mg/L of Ni(II) was 88% at 5% (w/v) adsorbent dosage and a further increase in sorbent dosage did not increase nickel removal [43]. The low rate of Ni(II) removal with the increase in sorbent dosage can be explained by sorbent particle agglomeration, which reduces the specific surface area and increases the diffusion path length [44]. In batch experiments, it was shown that with the increase in Ni(II) concentration in solution, its removal significantly decreases from 90% at Ni(II) concentration 10 mg/L to 28% at Ni(II) concentration in solution 100 mg/L. Thus, in the next experiment, the effluent was diluted two and twelve times and 0.5 or 1.0 g of sorbent was added to it. Ni(II) removal from the effluent diluted two times did not exceed 27%; however, a 12-fold effluent dilution resulted in the removal of 72% of Ni(II). Thus, it can be concluded that applied biosorbents are better suited for output wastewater which has been pretreated by traditional techniques or for wastewater with low Ni(II) concentrations. The efficiency of zinc and copper removal from the input raw wastewater into the treatment plant and output wastewater by *E. coli* biofilm placed on the zeolite was higher for wastewater treated previously by traditional techniques [4].

Besides the sorption capacity, one of the most important parameters of the biosorbent which determines its wide application is the cost. The price of zeolite in Russia varies from 78 to 916 USD per ton, depending on the supplier. The price of zeolite used in the present study was 380 USD per ton. Bacteria *Shewanella xiamenensis* do not require special growth conditions and the cost of mineral salt used for growth constituted 0.2 USD per liter of cultivation medium. The final price of the hybrid adsorbent would constitute 530 USD per ton of sorbent. The price of the studied hybrid adsorbent would be comparable with the price of ALO sorbent (200–600 USD per ton) [6], higher than the price of activated charcoal (approximately 100 USD per ton) [45] but lower than the price of single-walled carbon nanotubes, multiwalled carbon nanotubes, and granular activated carbon (90,000, 12,000, and 1000 USD per ton, respectively) [37]. It should be mentioned that the zeolite used in the present study was purchased from a commercial company in a small amount; its procurement from the producer in large quantities would significantly decrease the price.

## 5. Conclusions

Treatment of nickel-containing effluents using a hybrid adsorbent (*Shewanella xiamenensis* biofilm placed on zeolite) was tested. In synthetic effluents with different chemical compositions, the hybrid adsorbent showed a high affinity for cations, while anion adsorption did not occur due to the negative charge of the adsorbent surface at all studied pH values. The process of cation sorption was shown to be pH-dependent and the optimal pH for maximum metal ion removal (>70%) was 6.0. The experimental values at equilibrium were better adjusted to the Langmuir and Temkin isotherm models. The good fit of the pseudo-second-order and Elovich models suggested that the chemisorption and ion exchange were the main mechanisms of metal sorption. The decreased content of Na and K in the adsorbent was determined by NAA and supported this finding. The coefficients of determination obtained for equilibrium and kinetic models were in good agreement with the *R*^2^_adj_ values, indicating their applicability in explaining the experimentally obtained data. The thermodynamic data showed that the biosorption process was spontaneous and endothermic. An increase in biosorbent dosage from 0.5 to 2.0 g did not affect significantly the Ni(II) removal from real industrial effluent and it constituted 26%. However, a 12-fold dilution resulted in the removal of 72% of Ni(II) at a sorbent dosage of 1.0 g. The produced hybrid adsorbent can be considered as a sorbent with a high affinity for metal cations which can be applied for the treatment of diluted complex industrial effluents.

## Figures and Tables

**Table 1:** Metal concentrations in analyzed systems.

System	Concentration, mg/L						
	Ni	Cu	Zn	Sr	Fe	Cr	Mo
Ni(II)	10 ± 0.3	-	-	-	-	-	-
Ni(II)-Sr(II)-Cu(II)-Zn(II)	10 ± 0.1	1 ± 0.03	2 ± 0.008	5 ± 0.07	-	-	-
Ni(II)-Cr(VI)-Fe(III)	10 ± 0.3	-	-	-	5 ± 0.1	5 ± 0.12	-
Ni(II)-Zn(II)-Mo(VI)-Cu(II)	10 ± 0.2	5 ± 0.05	2 ± 0.03	5 ± 0.09	-	-	0.5 ± 0.003

**Table 2:** Chemical composition of industrial effluent.

Element	Sr	Mo	Cr	Ni	Cu	Zn	Fe	pH
Concentration, mg/L	0.3	0.002	0.005	125	0.025	0.072	0.004	6.0

**Table 3:** Elemental content of natural and bio-zeolite determined by neutron activation analysis NAA.

Element	Natural Zeolite	Hybrid Adsorbent	Element	Natural Zeolite	Hybrid Adsorbent
	Concentration, µg/g			Concentration, µg/g	
Na	13,000 ± 1000	9590 ± 300	Br	0.74 ± 0.02	0.98 ± 0.05
Mg	6570 ± 700	20,900 ± 1000	La	12.5 ± 0.5	40 ± 1.6
Al	67,700 ± 1300	60,200 ± 3000	U	3.97 ± 0.2	3.4 ± 0.12
Si	260,000 ± 2600	187,000 ± 1870	Sr	66.2 ± 6	n.d.
Cl	<81	334 ± 30	Rb	217 ± 20	n.d.
K	29,000 ± 2900	39,100 ± 3000	Sb	0.25 ± 0.01	n.d.
Sc	1.69 ± 0.06	n.d.	Ba	128 ± 10	n.d.
Ca	9850 ± 800	18,100 ± 1800	Cs	7.57 ± 0.2	n.d.
Ti	<556	964 ± 90	Ce	21.1 ± 2	n.d.
V	<3.4	8.3 ± 0.5	Eu	0.1 ± 0.01	n.d.
Mn	351 ± 17	107 ± 7.5	Gd	3.6 ± 0.2	n.d.
Ni	<8.49	95 ± 0.04	Tb	0.4 ± 0.01	n.d.
Fe	8560 ± 600	10,600 ± 640	Yb	2.1 ± 0.02	n.d.
Zn	62.9 ± 2.5	<12	Hf	6.3 ± 0.6	n.d.
As	1.28 ± 0.03	1.48 ± 0.05	Th	17.1 ± 1.0	n.d.

**Table 4:** Parameters calculated from the applied models.

		Ni(II)	Ni(II)-Cr(VI)-Fe(III)	Ni(II)-Sr(II)-Cu(II)-Zn(II)				Ni(II)-Zn(II)-Mo(VI)-Cu(II)		
	Metal	Ni	Ni	Ni	Zn	Cu	Sr	Ni	Cu	Zn
	*q_exp_, mg/g*	1	0.63	0.95	0.18	0.014	0.33	0.62	0.21	0.19
PFO	*q_e_, mg/g*	0.98	0.63	0.9	0.16	0.01	0.32	0.62	0.21	0.19
	*k*_1_ *, min^−1^*	0.06	0.04	0.06	0.06	0.05	0.05	0.06	0.14	0.05
	*R* ^2^	0.99	0.99	0.98	0.95	0.95	0.96	0.99	0.99	0.99
	R_adj_^2^	0.99	0.99	0.98	0.94	0.94	0.95	0.99	0.99	0.99
	*SSE, %*	0.09	0.08	0.39	0.08	0.02	0.93	0.2	0.03	0.9
PSO	*q_e_, mg/g*	1.07	0.73	0.98	0.19	0.014	0.36	0.67	0.22	0.21
	*k*_2_, *g/mg·min*	0.09	0.06	0.1	0.5	4.7	0.2	0.002	2	0.4
	*R* ^2^	0.99	0.99	0.99	0.96	0.96	0.93	0.99	0.99	0.99
	*R*_adj_^2^	0.99	0.99	0.98	0.96	0.95	0.92	0.99	0.99	0.99
	*SSE*	0.17	0.18	0.53	0.11	0.01	1.1	0.3	0.03	1.1
EM	*α*, *mg/g·min*	0.7	0.8	2.7	0.1	0.003	0.08	0.9	n.a	0.1
	*β*, *g/min*	6.6	7.2	5.8	35	407	15.6	0.01	n.a	31
	*R^2^*	0.98	0.97	0.97	0.98	0.93	0.9	0.98	n.a	0.97
	*R*_adj_^2^	0.98	0.96	0.96	0.96	0.94	0.88	0.97	0.99	0.96
IPM	*k_diff_*	0.07	0.06	0.06	0.01	0.001	0.02	0.4	0.01	0.01
	*C_i_*	0.25	0.1	0.2	0.04	0.003	0.07	1.7	0.08	0.05
	*R* ^2^	0.76	0.85	0.75	0.8	0.81	0.74	0.72	0.52	0.77
	*R*_adj_^2^	0.72	0.83	0.71	0.76	0.78	0.69	0.67	0.45	0.73

**Table 5:** The parameters of the applied adsorption isotherm models.

Model	Parameters	System			
		Ni(II)	Ni(II)-Cr(VI)-Fe(III)	Ni(II)-Sr(II)-Cu(II)-Zn(II)	Ni(II)-Zn(II)-Mo(VI)-Cu(II)
Langmuir	*q_m_*_,_ mg/g	3.9	3.7	3.6	1.98
	*b*, L/mg	0.04	0.03	0.04	0.05
	*R_L_*	0.2–0.7	0.2–0.8	0.19–0.7	0.17–0.68
	*R* ^2^	0.97	0.99	0.99	0.99
-	*R*_adj_^2^	0.97	0.99	0.99	0.99
Freundlich	*K_F_*, mg/g	0.53	0.4	0.53	0.33
	1/*n*	0.39	0.45	0.38	0.36
	*R* ^2^	0.92	0.98	0.96	0.98
-	*R*_adj_^2^	0.91	0.98	0.95	0.98
Temkin	*a_T_*, L/g	0.3	0.3	0.4	0.4
	*B*, kJ/mol	0.9	0.85	0.8	0.4
	*R* ^2^	0.96	0.99	0.99	0.99
-	*R*_adj_^2^	0.96	0.99	0.98	0.99

**Table 6:** Thermodynamic parameters for metal sorption on hybrid adsorbent.

System	Metal	*∆G*°, kJ/mol				*∆H*°, kJ/mol	*∆S*°, J/mol·K	*R* ^2^
		293 K	303 K	313 K	323 K			
Ni(II)	Ni	−10.4	–11.0	–11.5	–12.1	5.7	54.9	0.79
Ni(II)-Cr(VI)-Fe(III)	Ni	−10.6	–11.2	–11.8	–12.5	6.4	58	0.88
Nin(II)-Sr(II)-Cu(II)-Zn(II)	Ni	−10.6	–11.2	–11.7	–12.3	5.9	54.9	0.98
	Zn	−10.8	–11.3	–11.9	–12.4	5.0	54.0	0.99
	Sr	−9.6	–10.2	–10.7	–11.3	7.0	56.5	0.99
	Cu	−8.5	-9.2	−9.9	–10.5	11.4	68.2	0.98
Ni(II)-Cu(II)-Zn(II)-Mo(VI)	Ni	−11.1	−11.5	−12.0	–12.4	0.8	40.7	0.81
	Cu	−15.1	−15.8	−16.6	–17.4	7.8	60.6	0.92
	Zn	−9.9	−10.7	−11.4	–12.2	13	78.1	0.91

**Table 7:** Comparison of the sorption capacity toward Ni(II) of studied hybrid, as well as other sorbents.

Sorbent	*q_max_*, mg/g	Concentrations Range, mg/L	pH	Reference
Hybrid sorbent	3.2–3.6	10–100	6.0	Present study
Single-walled carbon nanotubes	47.85	10–80	7.0	[37]
Multiwalled carbon nanotubes	38.46	10–80	7.0	[37]
Granular activated carbon	26.39	10–80	7.0	[37]
*Escherichia coli* biofilm supported on zeolite NaY	9.85	10–100	6.0	[8]
*Arthrobacter viscosus* biofilm supported on zeolite	26.8	10–80	6.0	[9]
*Spirulina platensis*	13.4	5–100	4.0	[15]
*Calotropis procera*	15.75	5–500	3.0	[38]
*Carbonised coirpith*	62.5	20–40	5.0	[39]
Doum-palm seed coat	3.24	10–40	7.0	[36]
Zeolite	10	50–400	4.0	[30]

## Supplementary Materials

The following are available online at https://www.mdpi.com/1996-1944/13/19/4462/s1, Figure S1: The thermodynamic speciation of Ni(II) ions in Ni(II) system as a function of pH, Figure S2: The thermodynamic speciation of metal ions in Ni(II)-Cr(III)-Fe(III) system as a function, Figure S3: The thermodynamic speciation of metal ions in Ni(II)-Cu(II)-Sr(II)-Zn(II) system as a function of pH, Figure S4: The thermodynamic speciation of metal ions in Ni(II)-Zn(II)-Mo(VI)-Cu(II) system as a function of pH, Figure S5: lnKd versus 1/T.
