# Supplementary material for: Metal Removal from Nickel-Containing Effluents Using Mineral–Organic Hybrid Adsorbent
Source: Materials (Basel). 2020 Oct 8;13(19):4462. doi: 10.3390/ma13194462 (PMC7578995; doi:10.3390/ma13194462)
Supplement: Supplementary file 1 [file materials-13-04462-s001.pdf]

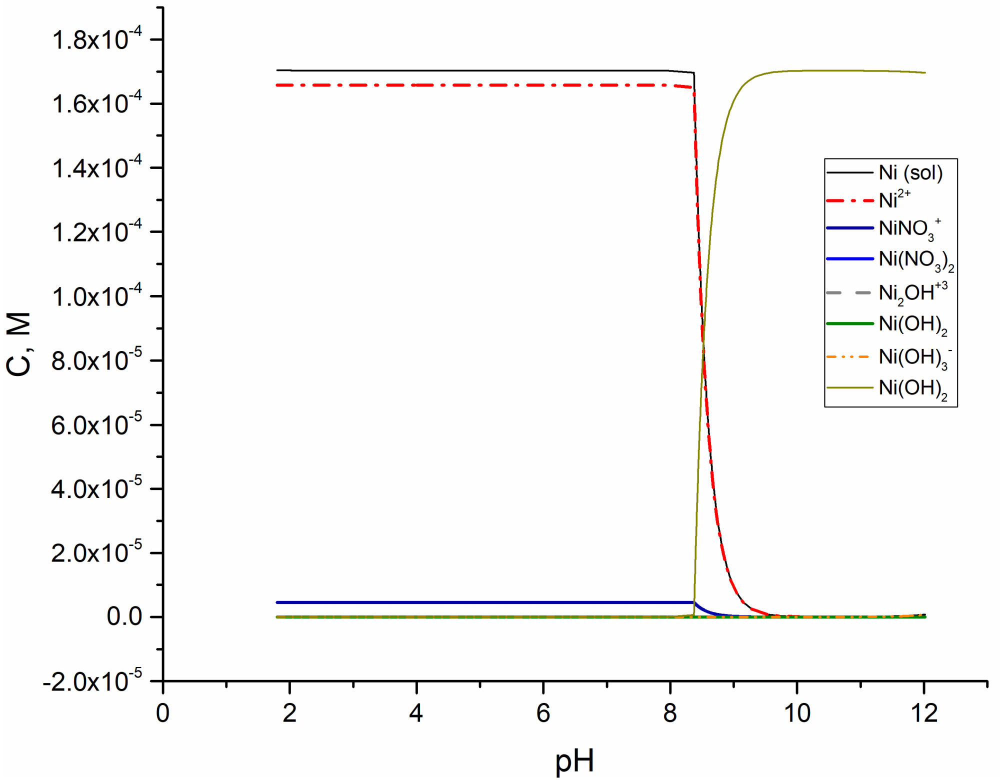

Figure S1. The thermodynamic speciation of Ni(II) ions in Ni(II) system as a function of pH.

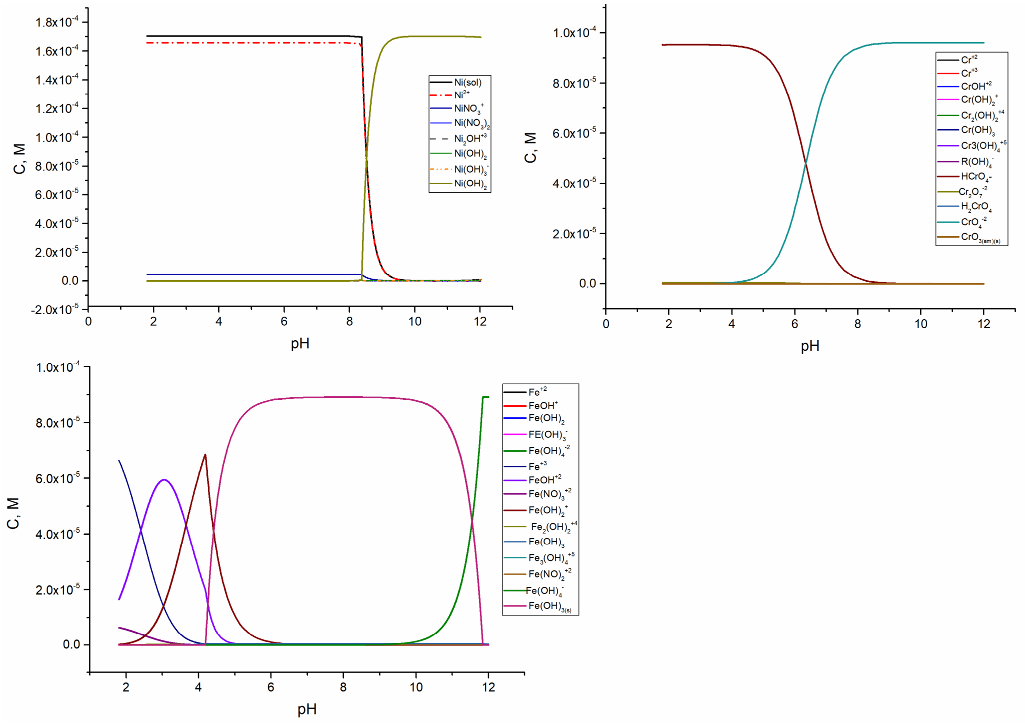

Figure S2. The thermodynamic speciation of metal ions in Ni(II)-Cr(III)-Fe(III) system as a function of pH.

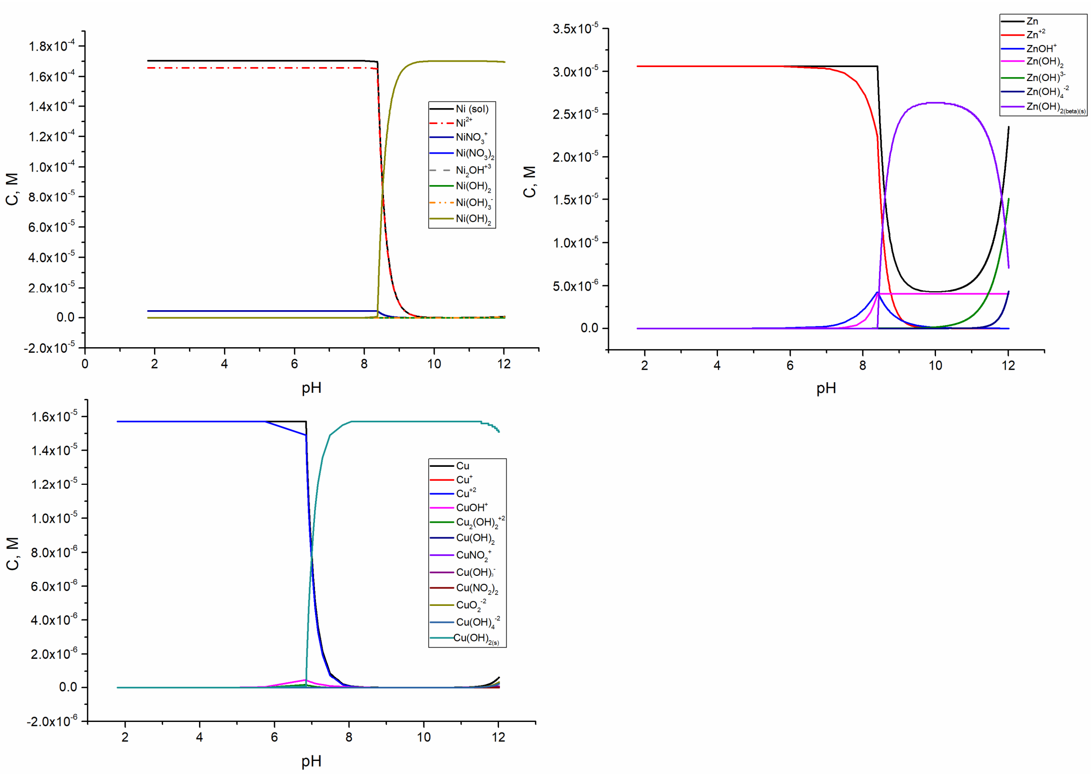

Figure S3. The thermodynamic speciation of metal ions in Ni(II)-Cu(II)-Sr(II)-Zn(II) system as a function of pH.

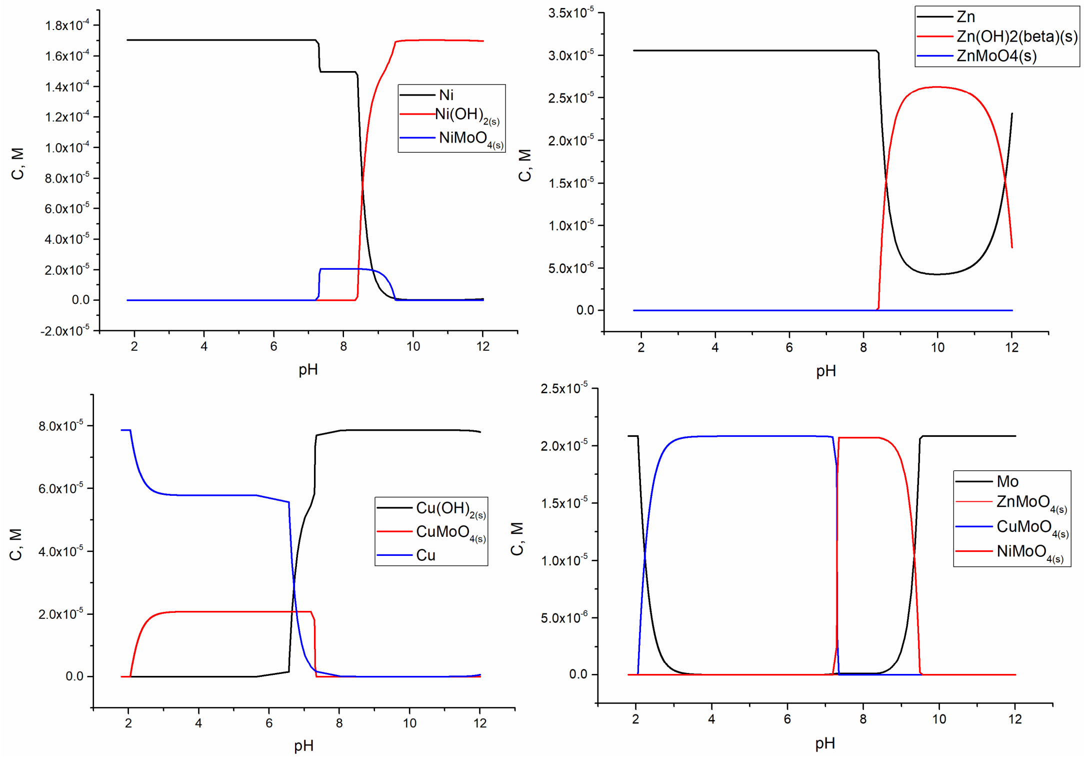

Figure S4. The thermodynamic speciation of metal ions in Ni(II)-Zn(II)-Mo(VI)-Cu(II) system as a function of pH.

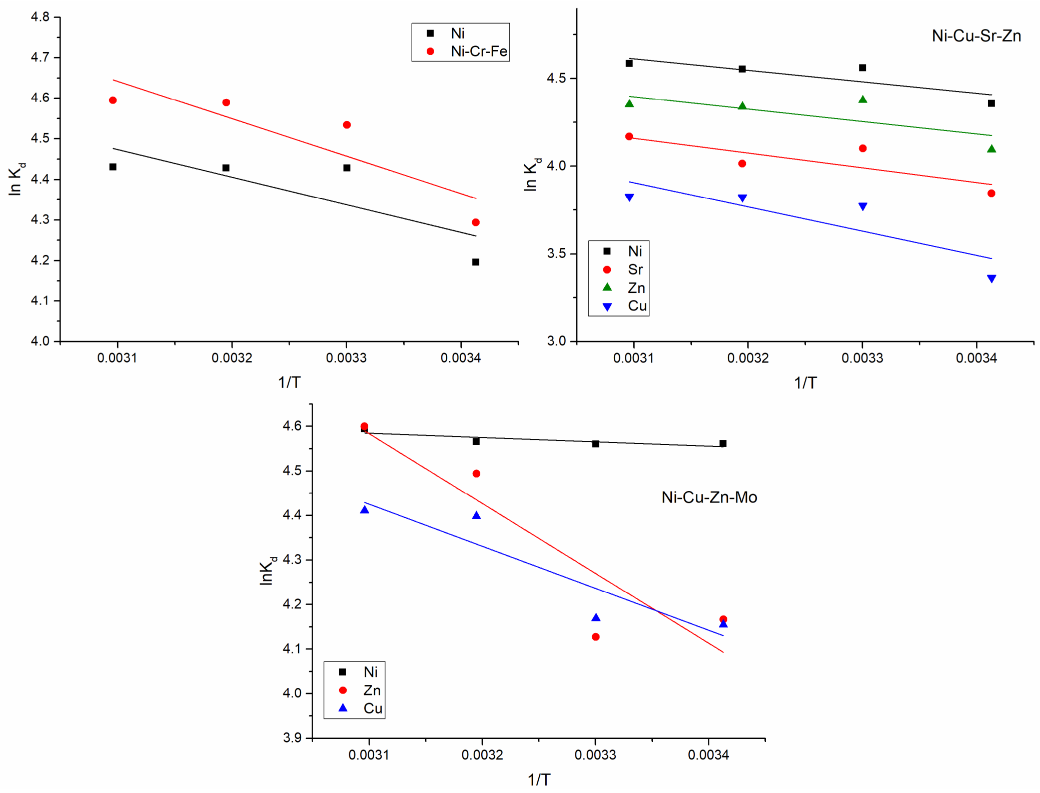

Figure S5. $\ln K_d$ versus 1/T.
